# An Attachable Electromagnetic Energy Harvester Driven Wireless Sensing System Demonstrating Milling-Processes and Cutter-Wear/Breakage-Condition Monitoring

**DOI:** 10.3390/s16030269

**Published:** 2016-02-23

**Authors:** Tien-Kan Chung, Po-Chen Yeh, Hao Lee, Cheng-Mao Lin, Chia-Yung Tseng, Wen-Tuan Lo, Chieh-Min Wang, Wen-Chin Wang, Chi-Jen Tu, Pei-Yuan Tasi, Jui-Wen Chang

**Affiliations:** 1 Department of Mechanical Engineering, National Chiao Tung University, Hsinchu 30010, Taiwan; scarletleaf.me02g@nctu.edu.tw (P.-C.Y.); peter800702.me98@g2.nctu.edu.tw (H.L.); stan86113@gmail.com (C.-M.L.); zxc351763x@yahoo.com.tw (C.-Y.T.); william123y@hotmail.com (W.-T.L.); chmin0217@gmail.com (C.-M.W.); 2 International College of Semiconductor Technology, National Chiao Tung University, Hsinchu 30010, Taiwan; 3 Precision Machinery Research Development Center, Taichung 40768, Taiwan; e8702@pmc.org.tw (W.-C.W.); tojijn@gmail.com (C.-J.T.); e9604@mail.pmc.org.tw (P.-Y.T.); e9419@pmc.org.tw (J.-W.C.)

**Keywords:** attachable, energy harvester, electromagnetic, wireless, vibration, sensing, milling monitoring, cutter condition, self-powered

## Abstract

An attachable electromagnetic-energy-harvester driven wireless vibration-sensing system for monitoring milling-processes and cutter-wear/breakage-conditions is demonstrated. The system includes an electromagnetic energy harvester, three single-axis Micro Electro-Mechanical Systems (MEMS) accelerometers, a wireless chip module, and corresponding circuits. The harvester consisting of magnets with a coil uses electromagnetic induction to harness mechanical energy produced by the rotating spindle in milling processes and consequently convert the harnessed energy to electrical output. The electrical output is rectified by the rectification circuit to power the accelerometers and wireless chip module. The harvester, circuits, accelerometer, and wireless chip are integrated as an energy-harvester driven wireless vibration-sensing system. Therefore, this completes a self-powered wireless vibration sensing system. For system testing, a numerical-controlled machining tool with various milling processes is used. According to the test results, the system is fully self-powered and able to successfully sense vibration in the milling processes. Furthermore, by analyzing the vibration signals (*i.e.*, through analyzing the electrical outputs of the accelerometers), criteria are successfully established for the system for real-time accurate simulations of the milling-processes and cutter-conditions (such as cutter-wear conditions and cutter-breaking occurrence). Due to these results, our approach can be applied to most milling and other machining machines in factories to realize more smart machining technologies.

## 1. Introduction

Recently, technologies for monitoring milling-processes and cutter-conditions [1,2,3,4,5,6,7,8] have become important for the machining industry to implement smart machining. According to the smart machining-monitoring technologies provided by leading machining companies [9,10,11,12], monitoring of milling-processes and cutter-conditions (such as cutter-wear conditions and cutter-breaking occurrence) can produce many significant benefits such as improving machining automation, preventing machining failures, decreasing machine downtime, and reducing maintenance costs. More recently, wireless sensing technology has been widely used in industrial and medical applications [13,14,15,16,17,18,19,20,21,22,23,24,25,26,27,28,29]. Specifically for the machining and manufacturing industries, researchers have demonstrated novel wireless sensing applications for real-time machining monitoring [19,20,21,22,23,24,25,26,27,28,29]. In these applications, wireless sensor nodes (or sensing systems) are used to detect and analyze critical factors (such as vibration and temperature of milling cutters) during milling. These real-time wireless sensing, analyzing, and monitoring tasks can prevent machining failures caused by over-cutting, chattering, and thermal-deformation during milling operations, and thereby increase the milling reliability (and still achieve the abovementioned benefits). However, replacing batteries is difficult when numerous sensor nodes (or sensing systems) are used. To solve this problem, energy-harvester-driven (*i.e.*, self-powered) wireless sensor nodes/systems are needed.

Nowadays, solar-cells (*i.e.*, as the energy harvester) are frequently used to power wireless sensor nodes/systems. However, these nodes/systems cannot perform normally when used for monitoring machining processes of machining machines located indoors in factories. Therefore, instead of using solar cells, researchers have utilized mechanical-vibration-based energy harvesters (such as piezoelectric [30,31,32,33,34,35], electromagnetic [30,31,36,37,38], electrostatic [39], and thermal energy harvesters [30,31,40,41]) to harness ambient vibration (such as lateral vibration and rotational vibration) to generate power for indoor applications [42]. For instant, piezoelectric energy harvesters which were specifically designed for harnessing lateral vibration and rotational vibration were demonstrated by Hung *et al*. [35] and Aktakka *et al*. [43], respectively. An electrostatic energy harvester which is specifically designed for harnessing lateral vibration was demonstrated by Beeby *et al.* [44] and Roundy *et al.* [45]. An electromagnetic energy harvester specifically designed for harnessing lateral vibration and rotational vibration was demonstrated by Glynne-Jones *et al.* [46] and Chang *et al.* [47], respectively. For the above energy harvesters, more applications can also be referenced from commercial energy-harvesting chip-platform solutions [42] and obtained from the literature. For instance, with some energy storage/management-circuits the piezoelectric energy harvesters presented by Hung *et al.* and Aktakka *et al.* can be used for structural monitoring applications which only require a small amount of energy to transmit a few data per day. The electrostatic energy harvesters presented by Beeby *et al.* can be used in an embedded sensing application for detecting waste gas emissions from cars. The electromagnetic energy harvesters presented by Glynne-Jones *et al.* and Chang *et al.* can be used for real-time wireless monitoring of machines.

Recently, some researchers used an electromagnetic energy harvester to harness mechanical energy from vibrations generated by a machining machine [48], and consequently intended to power wireless sensors for monitoring machining. Reference [48] presents a representative electromagnetic energy harvesting approach to power wireless sensing for machine monitoring. The harvester is well-designed to specifically harness the machine’s vibration in order to power the accelerometer for sensing. However, we found out that spindle rotation of the machine tools during machining can provide more mechanical energy than that provided by the machine’s vibration. Thus, an energy harvester specifically designed to harness spindle-rotation energy may be more suitable to power the wireless sensors. To address this issue, recently Chang and Lee demonstrated an electromagnetic energy harvesting approach for harnessing spindle-rotation energy [47]. They used an electromagnetic energy harvester to harness substantial mechanical energy from spindle rotation (instead of harnessing the smaller energy from machine vibration), and consequently demonstrated that the harnessed energy is sufficient to power a wireless sensor node/system for quality tests of as-manufactured spindles for spindle manufacturers before the spindles are sold to machining-machine manufacturers for further assembly. However, for machining and manufacturing societies, the real-time machining-processes monitoring is more critical and important than the quality testing of as-manufactured spindles. Furthermore, harvester-driven wireless sensor nodes or sensing systems for real-time condition monitoring of machining processes are still lacking in the literature to date. In addition, in Chang and Lee’s approach [47], the harvester and the wireless sensor nodes (or sensing systems) must be embedded inside the spindle, and thereby the spindle must be re-manufactured/modified. This not only causes spindle redesign and remanufacturing problems for different spindle manufacturers to adopt the wireless sensor nodes, but also may cause spindle compatibility problems for further assembly/installation into different machining machines. Moreover, in the general case of using wireless sensors network for milling-processes and/or cutter-condition monitoring of numerous machines distributed separately over a long distance in a factory, the wireless sensor nodes must achieve long-distance data transmission. However, the wireless sensor nodes’ protocols used by abovementioned researchers (RFID, Wifi, and Bluetooth) are not suitable for the required long-distance data transmission.

Thus, to solve these problems [*i.e.*, (I) absence of demonstration of the energy harvester driven wireless sensing systems to monitor real-time milling-processes and cutter-conditions; (II) re-design/remanufacturing/re-assembly problems of the spindles and the machines; and (III) insufficient data-transmitting distance of sensing nodes used in manufacturing factories], this paper demonstrates an novel attachable electromagnetic energy harvester driven wireless vibration sensing system to monitor milling-processes and cutter-conditions (such as cutter-wear conditions and cutter-breakage occurrence) in real-time. Because of its attachable features, spindle modifications are no longer needed as the sensing system is attached onto the machines (that is, the attachable feature is non-destructive for spindles) [49]. Moreover, the wireless sensing node used in this paper is a new developed powerful Zigbee-based node featuring low power consumption, long-distance signal transmission (up to 300 m), and capable of using numerous sensing nodes (maximum 65535 nodes) in a Wireless Sensors Network (WSN). Thus, the Zigbee-based node meets the requirements for using WSN to monitor numerous machines distributed separately over a long distance in a factory.

## 2. Design

**Figure 1:**
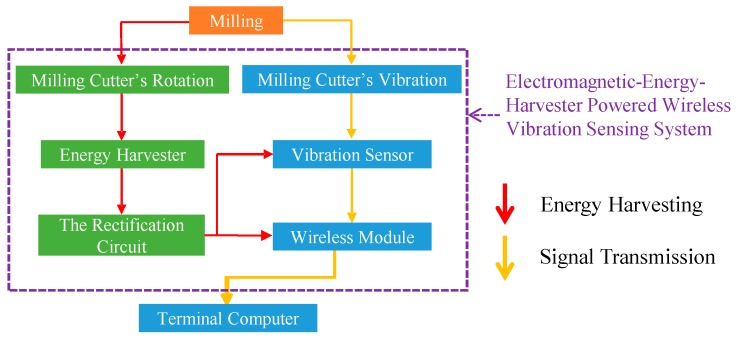
The energy harvesting and vibration-signal transmission approaches of the attachable electromagnetic energy harvester driven wireless vibration sensing system to monitor milling-processes and cutter-wear/breakage conditions. The red arrow-line indicates the energy-harvesting flow while the yellow arrow-line indicates the signal-transmission flow.

**Figure 2:**
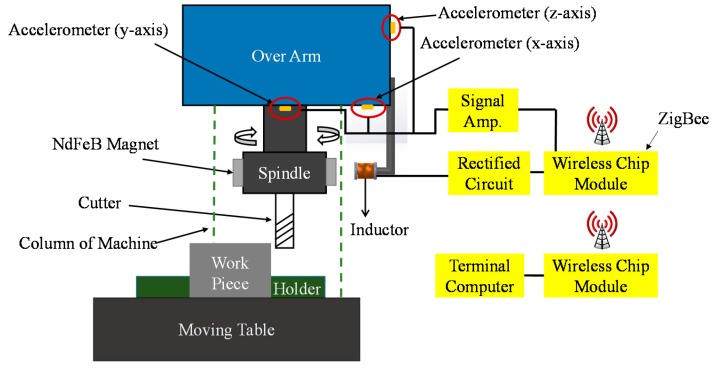
The front-view illustration of the testing setup (using the system to monitor milling-processes and cutter-wear/breakage conditions).

The energy harvesting and vibration-signal transmission approaches of our proposed sensing system are shown in Figure 1. According to the approaches, the corresponding illustration of the testing setup is shown in Figure 2. As shown in Figure 1 and Figure 2, the system includes an electromagnetic energy harvester, three MEMS single-axis accelerometers, a wireless chip module, and corresponding circuits. The harvester consists of four permanent NdFeB magnets (fixed on the spindle) and an inductance (fixed on the machine). According to the energy-harvesting process indicated by the red arrow-lines in the flowchart in Figure 1 and the testing setup in Figure 2, the magnets are rotated with the spindle during milling and consequently a cycled magnetic flux is produced to the inductance. 

Because of the electromagnetic induction, alternating voltages are induced in the inductance by the cycled magnetic flux (*i.e.*, it produces an alternating power output). Therefore, the mechanical-energy of cutter rotation during milling operations is harnessed and simultaneously converted into electrical energy. Furthermore, by the rectification circuit (*i.e.*, modified bridge-circuits with voltage regulators), the converted electrical energy can sufficiently operate the MEMS accelerometers and wireless chip module. Due to this, a self-powered wireless vibration sensing system is achieved. In addition, according to the vibrational signal transmission flow indicated by the yellow arrow-lines in the flow-chat in Figure 1 and the testing setup in Figure 2, when the cutter contacts the work piece during milling, vibrations are generated. Consequently, the accelerometers detect the vibration. Subsequently, the wireless chip module transmits the accelerometers’ vibration signals to a receiver in a terminal computer. After the terminal computer receives the signals, the signals are analyzed through the criteria to indicate the actual real-time milling and cutter-wear/breaking conditions. This achieves a real-time milling-processes and cutter-wear/breaking conditions monitoring.

## 3. Fabrication and Testing

**Figure 3:**
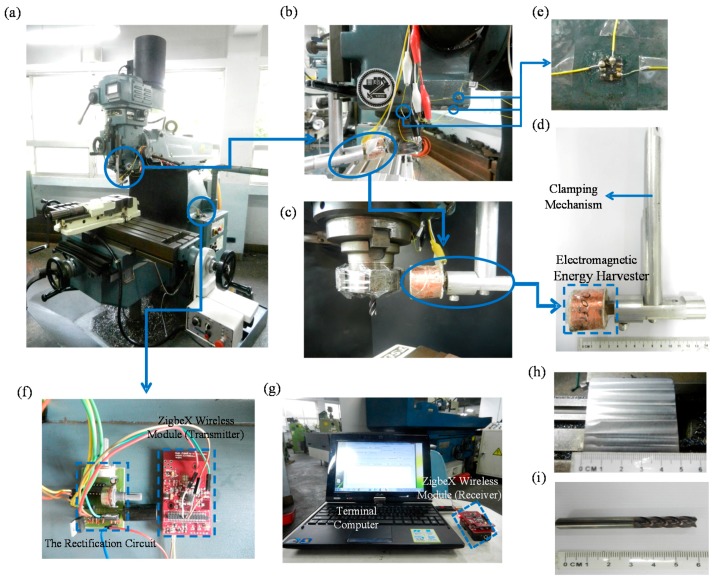
Photographs of the fabrication and testing: (**a**) test setup; (**b**) and (**c**) enlarged view of the locations of the electromagnetic energy harvester and MEMS accelerometers; (**d**) and (**e**) enlarged view of the harvester and accelerometers; (**f**) enlarged view of both the wireless chip module (used as the transmitter) and the rectification circuit; (**g**) wireless chip module (used as the receiver) and the terminal computer; (**h**) top view of the work piece; (**i**) milling cutter.

According to the illustration in Figure 2, a photograph of the corresponding testing setup is shown in Figure 3a. In Figure 3a, a numerically-controlled milling machine (QJM-QB, Quickjet, Taichung, Taiwan) is used to test our system. In Figure 3b,c, an electromagnetic inductance (which is fixed on the machine) and four NdFeB hard magnets (which is fixed on the spindle) are used to fabricate the energy harvester. The dimensions of the fabricated harvester are shown in Figure 3d. As shown in Figure 3b, three MEMS single-axis accelerometers (used as x-, y- and z-axial accelerometers) are fixed at locations (owing to a relative maximum vibration-response) on the machine’s over arm in order to detect the x-, y-, z-axial vibration produced by the cutter during milling. Because milling processes typically and mainly generate low-frequency vibrations, we chose accelerometers (AXDL 103, Analog Device, Norwood, MA, USA, shown in Figure 3e) with great sensitivit in order to successfully detect the low-frequency vibrations. The location (owing the relative maximum vibration-response) is determined as follows: first, the accelerometers are connected to an oscilloscope and consequently the accelerometers are placed on the surface of the overarm of the machine to directly measure vibrations from machines. We changed the location of the accelerometers (*i.e.*, scanning the surface) and continuously measured the vibration. After several cycles of scanning and measuring, the amplitudes of the vibration signals at different locations are compared to determine which location has a relatively maximum vibration response on the surface of the over arm. After placing the accelerometers, milling-induced vibrations can be detected by the accelerometers. To distinguish the sensor’s signals from noise, the signals are amplified by a differential amplification-circuit before transmitting to the wireless chip module (the corresponding printed circuit module of the differential amplification-circuit is shown in Figure 3f). The rectification circuit (as shown on the left-hand side in Figure 3f) is customized for our system and fabricated on print-circuit boards as compact circuit-modules in order to be easily attached on the machine.

**Figure 4:**
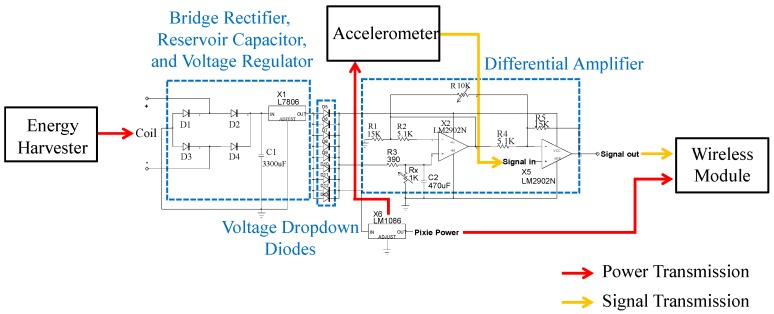
Circuit diagram of the rectification circuits.

The details of the corresponding circuit diagram are shown in Figure 4. To reduce the power consumption on transmitting the vibrational signals from the wireless chip module to the terminal computer, a low-power-consumption ZigBeX wireless chip module using the IEEE 802.15.4 communication protocol (Pixie Lite, FlexiPanel, London, UK) is used, as shown in Figure 3f. The module used a ZigBee-ready RF-antenna (CC2420, Texas Instruments, Dallas, TX, USA) for wireless data-transmitting/receiving and an ATmega128L microcontroller (Atmel Corporation, San Jose, CA, USA) for both signal processing and control of the RF transceiver. The total transmission rate of the wireless-chip module for 3-axial vibration sensing is 450 vibrational-signal data per second (*i.e.*, for single-axial vibration sensing: 150 vibrational signal-data per second) sending from the wireless chip module to the ZigBee receiver which is connected with the terminal computer as shown in Figure 3g. Therefore, the sampling rate which depends on the transmission rate is 150 Hz. For the preprocessing of the signal-data, we use the differential amplifier (see the circuit diagram shown in Figure 4) to eliminate the voltage bias of each accelerometer (voltage bias is around 1 to 1.5 V due to the 1 g gravity of Earth) and to amplify the vibration-signals properly to be easily observed when transmitting signals to the wireless receiver (*i.e.*, the wireless-chip module on the terminal computer side). Finally, the energy harvester, MEMS accelerometers, and wireless chip module are integrated as the attachable energy-harvester driven wireless vibration-sensing system. After integration, the system is tested as following.

For all tests, the wireless chip module and the rectification circuit are attached on a lateral side of the column of the milling machine, as shown in Figure 3f. Due to this, the module and circuits are able to avoid swarf influence which is produced by milling. The work piece is a JIS SS41 carbon-steel thick plate as shown in Figure 3h. The cutter (GSX4C-2.5D, Nachi-Fujikoshi Corp., Toyama, Japan) is an 8-mm tungsten carbide end-mill cutter with four teeth as shown in Figure 3i. In milling processes, the power provided from the harvester must be sufficient to drive the whole system. That is, the generated power (from the harvester) must be larger than the power consumption of the system. Due to this, the spindle’s rotational speed is gradually increased from static status in order to obtain the minimum rotational-speed of the spindle to cause the harvester to achieve the minimum power requirement to drive the system. According to the power feature of the Zigbee wireless chip module, the minimum power requirement (*i.e.*, power consumption) of the system are able to be measured while both the supplied power actually activates the Zigbee wireless chip module for data transmission (which can be confirmed from the power-indicator of the module) and the supplied voltage actually activates the accelerometers for vibration sensing (which can be checked by measuring voltage outputs of the accelerometers through utilizing an oscilloscope). In addition, the power generation and power consumption of the system at this moment can be measured and consequently used for further experimental-based power-budget analysis which is important design reference for applying the system to other machining machines in the future. Finally, the system is tested at this rotational-speed in several representative milling-processes.

## 4. Results and Discussion

**Figure 5:**
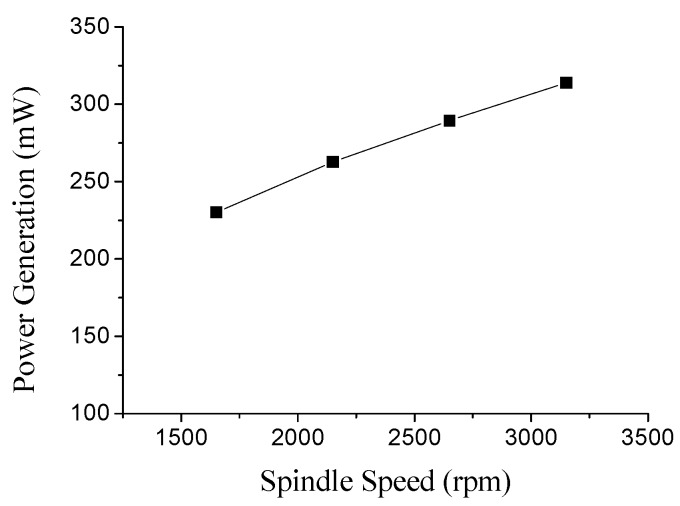
Power generation from the energy harvester at different spindle’s speeds.

According to the test results of the minimum power requirement for driving the system, a spindle operating at a rotational-speed of 1650 rpm can produce the minimum mechanical energy harnessed by the energy harvester to power up the system (*i.e.*, can match the minimum power requirement indicated by the Zigbee wireless chip module and the 3.3 V minimum voltage requirement of both the wireless chip module and the accelerometers). That is, operating the spindle at any rotational-speed higher than 1650 rpm guarantees to activate the harvester to generate sufficient power and voltage to drive the system. Furthermore, energy-harvesting outputs at different operating speeds are also measured. The measured results are shown in Figure 5, where as the spindle is operated at rotational speeds of 1650, 2150, 2650 and 3150 rpm, the power generated by the energy harvester connecting abovementioned load-resistance with equivalent impedance is 230.14, 262.74, 289.43 and 313.88 mW, respectively. These power generation results show that (and also prove our claim that) the power generation of the energy harvester is increased as the spindle’s rotational speed is increased. The above power-measurement of the energy harvester is described below. An electric meter (CIE-3122B, Chung Instrument Electronics Industrial, New Taipei City, Taiwan) is used to measure the voltage and current generated by the harvester which is connected to the other part of the rectification circuit (*i.e.*, connected to an equivalent impedance of the rectification circuit as a close-loop). Thus, the harvester can be regarded as connecting to a load resistance with equivalent impedance [35]. Furthermore, the experimental set up to measure the consumed power of each element (including the wireless-chip module) is described in following. For each component, the electrical meter is used to measure the voltage crossing each element and the corresponding current passing through each component. Based on the measured voltage and current, we calculated the power consumption of each component using the equation P = I*V, where P, I, V stands for power consumption, current, and voltage of each component. Finally, we summarize these measured and calculated results in the Table 1. In Table 1, the power consumption of the LM7806 and LM1086 voltage regulators, voltage dropdown diodes, and differential amplifier are 60.66, 54.47, 17.95 and 2.14 mW respectively. Three ADXL-103 accelerometers require a total operational power of 4.47 mW. To drive the ZigbeX wireless chip module for wireless data transmission, a power of 83.66 mW is needed. Thus, to sum up, the overall power consumption to operate the whole system (including all components) is 223.35 mW. Simultaneously, Table 1 also shows the measured energy harvesting (power generation). In Table 1, the power generated by the harvester is 230.14 mW. When comparing the generated power (230.14 mW) and the total consumed power (223.35 mW), the slight discrepancy is due to the thermal power dissipation caused by unavoidable small current leakage of the circuits. Due to these results, the power budget analysis reconfirms that operating the machine’s spindle at this rotational speed guarantees to cause the harvester to supply sufficient power to drive the system, which is in consistent with the verification result of the Zigbee’s power indicating-function. Up on this, the verified approach of the power-budget analysis can be used as the system is applied to monitor other different machining machines and as the system is expanded to include power management circuits for more complicated WSN machining-monitoring applications. Finally, we have to clarify that no power management solution is used in our system, because the system does not utilize any energy storage or energy-storage function (due to the large amount of energy produced by the electromagnetic energy harvester). Because large amount of energy produced by the electromagnetic energy harvester are able to sufficiently drive the system to perform most wireless sensing demonstrations. Therefore, using the power management solution is not necessary for our system in this study but will be important for continuously developing the system for other case-studies in the future [50].

In addition, we note that operating the spindle at 1650 rpm also provides some benefits because this low speed not only is in the range of typical starting speeds (1213–1819 rpm) specific for our cutter and work-piece under general milling operations (see Table 24-1 “suggested starting feeds and speeds using high sped steel and carbide cutters”) [51] for testing the general performance of the system, but also is easily to be observed in frequency spectrum analysis rather than observing other frequencies with many digits after decimal point [Note: the spindle’s rotational speed of 1650 rpm is equal to 27.5 rps; because the cutter has four teeth, thereby the milling frequency (*i.e.*, the speed times the number of the teeth; 27.5 rps × 4) is 110 Hz]. Finally, because the harnessed energy is increased as the rotational speed is increased, testing the system at low speed (rather than high speed) is more important to validate the design concept. Furthermore, because the spindle’s rotational speed for milling processes is 1650 rpm and the diameter of the four-teeth cutter is 8-mm, the corresponding cutting speed is calculated as 41.46 m/min (through the equation V = πDN/1000, where D is the diameter of the cutter and N is the spindle’s rotational speed). In addition, the milling process is operated with no cutting fluid. Due to these milling conditions, the depth of cut is determined to set as 2 mm. Furthermore, the milling direction is along the x-axial direction. Therefore, the feed rate (which is estimated as the milled x-axial length dividing the milling duration) is 283.55 mm/min. These rotational-speed-dependent milling parameters/conditions for the milling-processes monitoring are summarized in Table 2.

**Figure 6:**
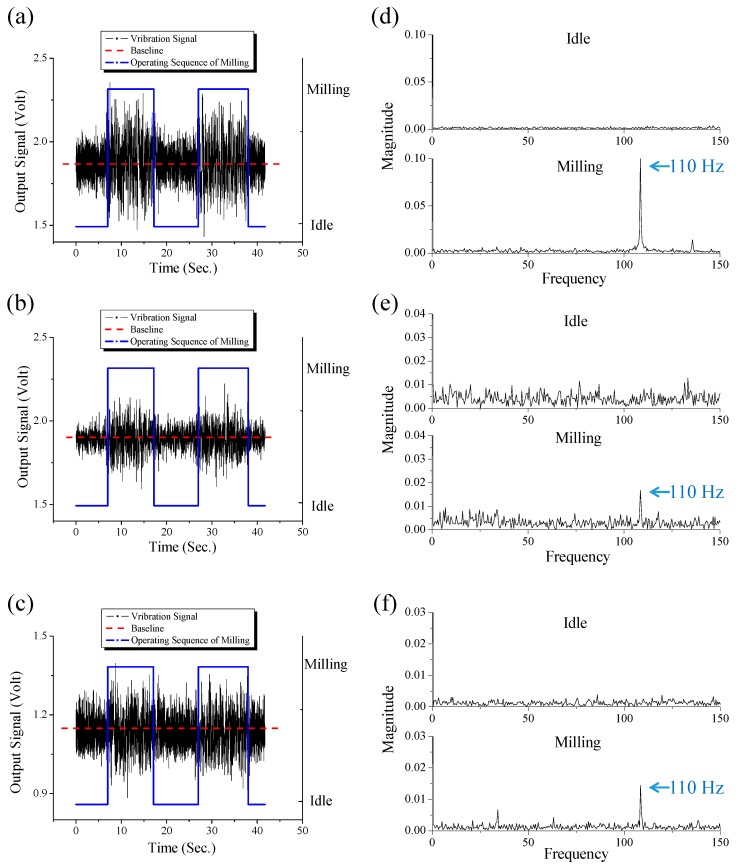
The (**a**) x-axial; (**b**) y-axial; (**c**) z-axial time-domain-based vibration signals received by using the system in milling processes. The corresponding (**d**) x-axial (**e**) y-axial (**f**) z-axial frequency-domain-based vibration signals for “idle” and “milling” statuses in milling processes. These frequency-domain results are obtained by applying Fast-Fourier Transform to (**a**), (**b**), and (**c**), respectively.

Figure 6 shows the test results of the system used for milling-processes monitoring with above-mentioned milling conditions. As shown in Figure 6, the actual milling sequence of “idle, milling, idle, milling, and then idle” is indicated by the blue line. Each red line indicates each base-limit of the vibration signals of each capacitive MEMS accelerometer (*i.e.*, each base-limit represents the voltage bias to active each accelerometer). Thus, the output signals will oscillate above and below the base line. Each black line (fitting the data) represents each axial output signals read from the terminal computer. When the work piece is milled by the cutter, the system obtained 3-axial vibration signals as following: (I) x-axial vibrational signals are voltage outputs higher than 2.11 V or lower than 1.64 V (Figure 6a); (II) y-axial vibrational signals are voltage outputs higher than 2.05 V or lower than 1.75 V (Figure 6b); (III) z-axial vibrational signals are voltage outputs higher than 1.28 V or lower than 0.99 V (Figure 6c). When comparing Figure 6a–c, the high and low limits of the voltage range of the z-axial signals obtained from the z-axial accelerometer are lower than those of x- and y-axial signals obtained from the x- and y-axial accelerometers. This is caused by the gravity effect subjected to the z-axial accelerometer, according to both setting location and sensing mechanism of the accelerometers. Through these lines, the signals representing the milling and idle statuses of the milling processes can be initially and roughly distinguished.

In addition, the results in Figure 6a–c from the time domain were transformed to the frequency domain by using a Fast Fourier Transform (FFT) (Note: we used an oscilloscope (which is connected to accelerometers) to directly receive vibration signals from the accelerometers, and consequently we applied FFT for the signals on another computer). The transformed results in frequency domain are shown in Figure 6d–f, where the results indicate that the frequency of vibration signals produced during the milling processes is 110 Hz. This confirms that the vibration signals are actually produced from the milling processes. These results perfectly verify that the vibration during the milling oscillates the accelerometers and consequently produces the oscillated voltage outputs in 110 Hz. However, certain oscillated voltage outputs during milling are not significantly higher or lower than the base limit. To investigate this, an oscilloscope is used to individually analyze the oscillated voltage outputs (as a reference to our sensing system). The oscilloscope (sampling frequency of 2500 Hz) is connected to accelerometers to directly receive vibration signals from the accelerometers (*i.e.*, measure the voltage output of accelerometers) during the status of milling processes. The analyzed results show that only approximate 30% of the vibration signals are in the voltage range considered as “idle”. This indicates that more than 70% of the voltage signals actually represent the vibration. This confirms that our system can successfully detect most vibration (more than 70%) in milling processes. Because the oscillated voltage outputs have more than 70% probability to actually indicate the milling status, the criterion can be modified to increase the probability (*i.e.*, improve the accuracy). Because the fact that some data obtained in milling status have smaller difference than the data obtained in idle status, only one voltage-amplitude datum (in Figure 6) is used in the criterion for direct determination of the real-time status of milling/idle sometimes may cause errors in certain cases. Therefore, using a consequential data set for the criterion would indicate the actual milling sequence/status more accurately. Due to this, through analyzing the vibration data received from accelerometers, the criterion is modified to be capable of accurately indicating and simulating milling processes with different operating sequences. That is critical to achieve an accurate real-time milling-processes monitoring.

**Figure 7:**
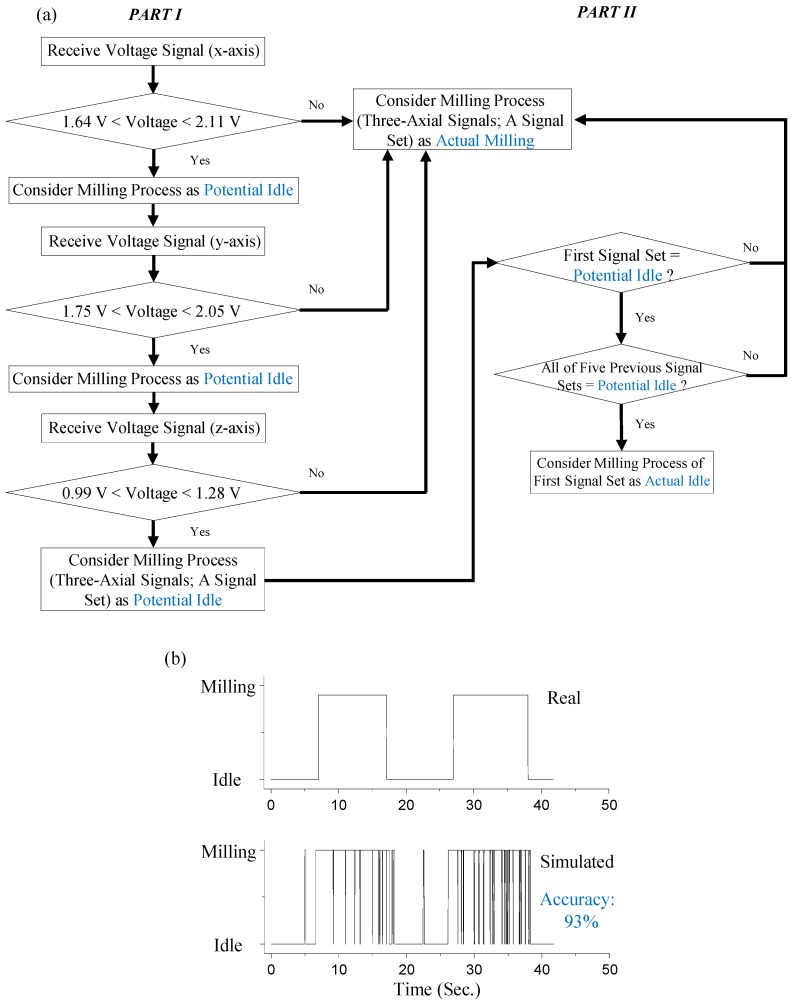
Using criterion for simulating milling status/sequence and corresponding results: (**a**) flow chart of the criterion, (**b**) comparing simulated status/sequence to real status/sequence.

According to the abovementioned issue, the modified criterion is shown in Figure 7a. Through the criteria, both the actual and the simulated (criterion-based) milling status/sequences are shown in Figure 7b. The first part of the criterion in Figure 7a is described below. First, three axial data (*i.e.*, x-, y-, and z- axial signal received by the accelerometers at the same time) are checked as a signal set. In the signal set, if the received x-axial signal (from the x-axis accelerometer) is either higher than 2.11 V or lower than 1.64 V, the signal set indicates the “Actual Milling” status is considered. On the contrary, if the received signal is in the range of 1.64 V to 2.11 V, same approach is continuously used to check the y-axial signal (received from the y-axial accelerometer). If the received y-axial signal is higher than 2.05 V or lowers than 1.75 V, the signal set indicates the “Actual Milling” status. Otherwise, same approach is continuously used to check the z-axial signal received (from the z-axial accelerometer). If the received z-axial signal is higher than 1.28 V or lower than 0.99 V, the signal set indicates the “Actual Milling” status. Otherwise it is considered as a “Potential Idle” status (*i.e.*, as three axial data are continuously checked in a roll and only if NONE of the three axial data indicates the “Actual Milling” status, the current status of the signal set is considered as an “Potential Idle”). Furthermore, the final official status of these “Actual Milling” and “Potential Idle” statuses are determined through the second part of the criterion. The flow chart of the second part of the criterion (right-hand side of the Figure 7b) is described as follows: if the first signal-set (first received 3-axial data at the same time as the set) indicates the “Potential Idle”, the status of the previous signal-set (previous received 3-axial data at the same time as the set) is consequently checked. If the status of the previous signal-set is also checked as indicating the “Potential Idle”, the status of one signal-set received before the previous signal-set is consequently checked. The checking process is repeated until total six continuous signal-sets (*i.e.*, one original signal-set and five previous signal-sets) are all checked. Finally, if all of the six signal-sets’ statuses are “Potential Idle”, the first signal set’s status is officially determined as “Actual Idle”. However, if any of six signal-sets’ statuses is not “Potential Idle”, the status is determined as “Actual Milling”. Through the second part of the criterion, the accuracy of indicating actual operating-sequence is increased from 70% to at least 93% (as shown in Figure 7b). That is, the chance of the false-indicating/detection is significantly reduced through using the criterion.

**Figure 8:**
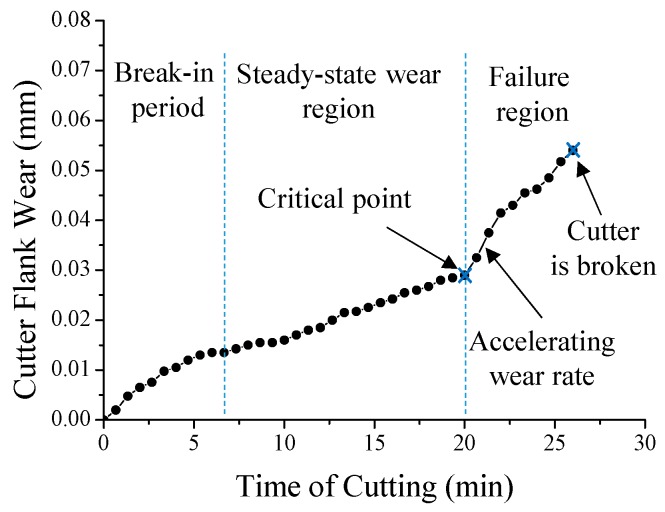
The tool wear curve (measuring the wear until the cutter is broken).

In addition to the real-time milling-process simulation, the system can detect cutter wear problems which frequently occur during a long-duration accumulated operation in general milling processes. That is, the system can characterize the wear conditions of the cutter by investigating the differences between the vibration signals generated by the cutter under different milling conditions. For the wear monitoring tests, the general milling parameters/conditions are the same as those of the real-time simulation of the milling process. According to these milling conditions, a complete tool wear measurement is conducted. The measuring method is described as follows: a micrometer (Mitutoyo micrometer series 103 with the accuracy of ±0.002 mm, Mitutoyo Corporation, Sakado, Japan) is used to measure the reduction of the diameter of the cutter every 40 s during a continuous milling operation. For each measurement, because the cutter has four teeth, the diameter of the cutter from different pairs of teeth is measured four times and consequently averaged. The averaged change of the diameter indicates the wear value of this measurement. The measurement results are shown in Figure 8. The total milling time for this cutter-wear experiment is 26 min because the cutter is broken after 26 min milling. The amount of the tool wear in the last measurement (*i.e.*, 25 min) before cutter breakage is measured as 0.054 mm. Based on the results, a corresponding tool-wear curve (*i.e.*, tool flank wear as function of cutting time) [52] is plotted, also shown in Figure 8.

**Figure 9:**
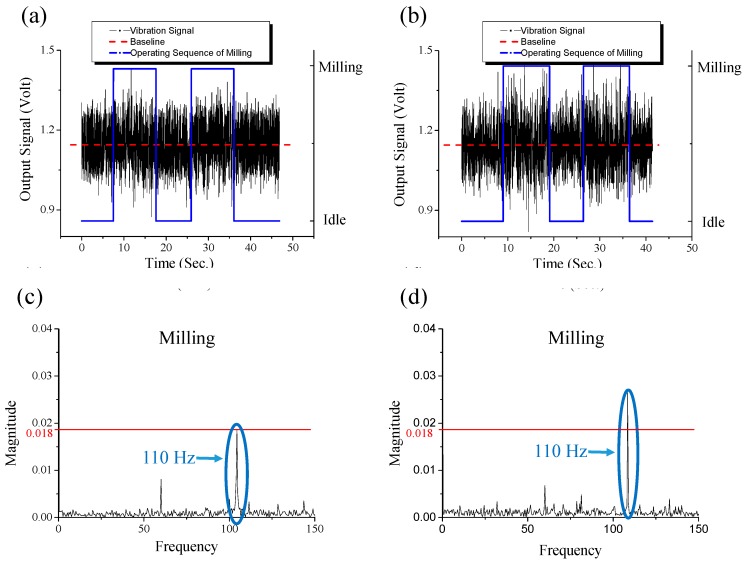
The time-domain z-axial vibrational signals at (**a**) critical point; after 20 min milling; (**b**) the last point before cutter-breakage after 25 min of milling. The corresponding frequency-domain signals in “milling” status at (**c**) critical point; after 20 min milling, (**d**) the last point before cutter-breakage; after 25 min milling.

**Figure 10:**
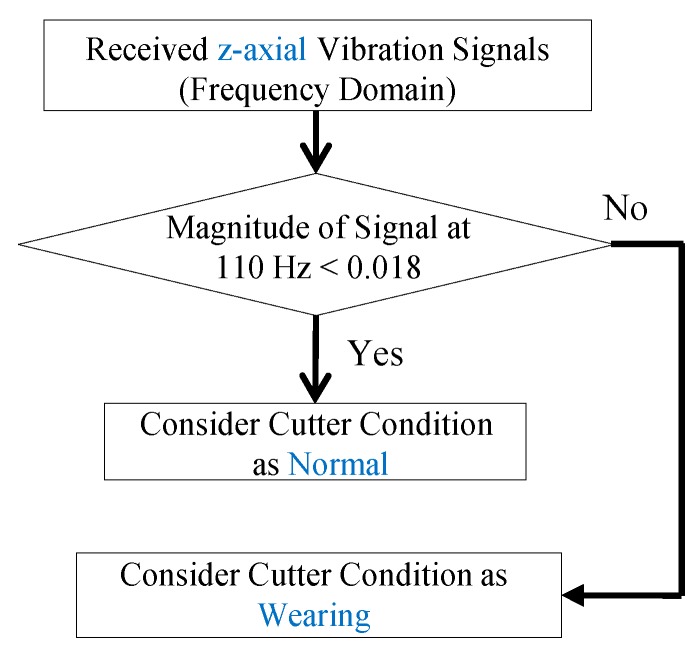
The criterion for monitoring the cutter’s wear condition during milling.

The curve represents typical three stages of tool life [52]. The three stages are the break-in period (with rapid initial wear), steady-state wear region (with uniform wear rate), and failure region (with accelerating wear rate). Based on the curve, the critical point between the steady-state wear region and the failure region (*i.e.*, when the slope of the curve dramatically changes at the end of the steady-state wear region) is exactly the measured point when the cutter completes its 20 min milling. Furthermore, the time-domain and frequency-domain z-axial vibrational signals at the critical point (after 20 min milling) and the last point before cutter is broken (after 25 min milling) are compared, as shown in Figure 9. In Figure 9c,d, the magnitude of the 110 Hz peak is increased as the wear of the cutter grows. Therefore, the magnitude (0.018) of the 110 Hz peak at critical point is used to establish a criterion (in Figure 10) to check whether the cutter’s wear status is getting into the failure region (*i.e.*, whether the wear rate is changing from uniform rate to accelerating rate). Through the criterion, the tool-wearing condition of the cutter is successfully monitored. By the monitoring, the severe wear tool is able to be replaced in the early stage when it just reaches the failure region.

**Figure 11:**
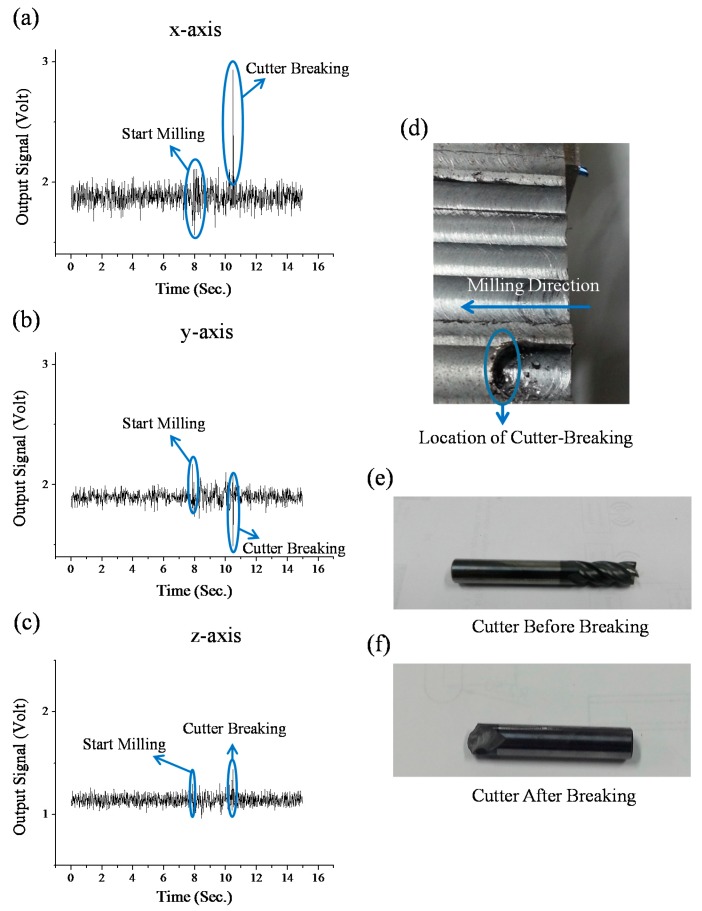
The results of the cutter-breaking monitoring test: (**a**–**c**) Three axial vibration signals received from the attachable electromagnetic-energy-harvester-driven wireless vibration-sensing system while the cutter is breaking during the milling; (**d**) The photograph of the milled surface of the work piece. (**e**,**f**) cutter before and after breaking.

**Figure 12:**
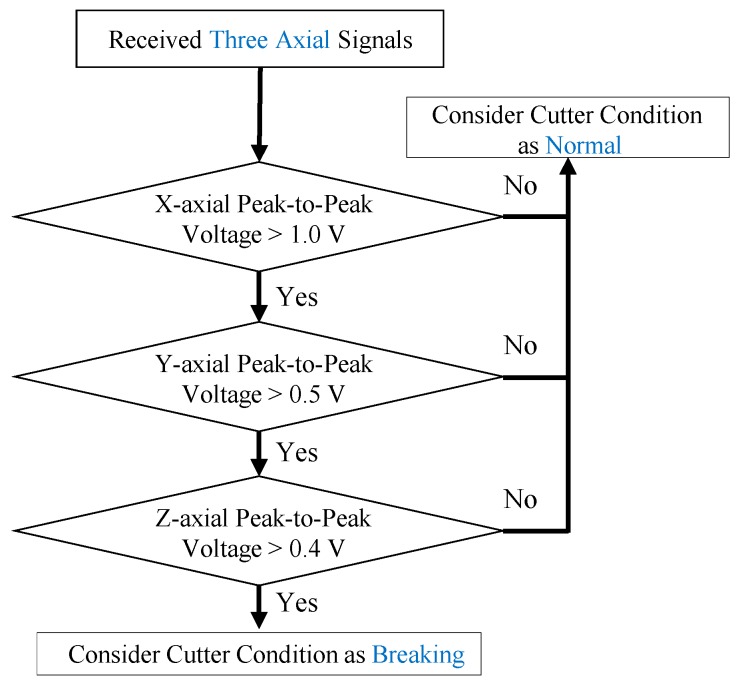
The criterion for monitoring the cutter breaking during milling.

Moreover, the sensing system can also detect a sudden cutter breakage which occurs frequently in many unexpected milling conditions (such as overloaded milling, overcutting, and severe cutter-wear). In addition, when an unexpected impact-accident occurs during the milling, the cutter may be broken suddenly rather than experience gradual cutter-wear. Due to these facts, monitoring cutter-breakage can immediately reduce machine down-time and thus is an important option for users. For the cutter-breakage testing, the general milling conditions are the same as other tests in the paper. However, the cutter for this testing is prepared from a severe damaged cutter after long-duration operation (*i.e.*, the cutter’s one tooth has a milling-produced geometric discontinuity). During the cutter-breakage test, the cutter is used to continuously mill the work piece until the cutter breaks. The continuous milling produces continuous three axial vibrational signals as shown in Figure 11a–c. The photographs of the milled surface of the work piece are shown in Figure 11d. In addition, the cutter before and after breaking is shown in Figure 11e,f, respectively. In Figure 11a–c, at the moment when the cutter is breaking during milling, an instantaneous large mechanical-impact is produced. The impact induces an instantaneous ultra-large voltage peak in the x-axial vibrational signals, and a relatively large voltage peak in both the y-axial and z-axial vibration signals (which is reasonable because the milling direction is toward the x-axial direction). After the cutter is broken, the vibrational signals immediately go back to the original before-breaking status. Because of the fact that the three axial voltage peaks are instantaneously produced while the cutter is breaking, the real-time cutter-breakage can be successfully monitored by the system by measuring the magnitude of the voltage peaks. According to this, a criterion (as shown in Figure 12) to detect the cutter-breakage during milling is further established. As shown in Figure 11, if the peak-to-peak voltage of any x-axial signal received from the x-axial accelerometer is lower than 1.0 V, the cutter condition is considered as a “Normal” status. Otherwise, the same approach is continuously used to check the y-axial signal received from the y-axial accelerometer. If the received y-axial signal is lower than 0.5 V, the cutter condition is considered as the “Normal” status. Otherwise, the same approach is continuously used to check the z-axial signal received from the z-axial accelerometer. If the received z-axial signal is lower than 0.4 V, the cutter condition is considered in the “Normal” status. Otherwise, we continuously use the same approach to check the z-axial signal received from the z-axial accelerometer. If the received z-axial signal is lower than 0.4 V, the cutter condition is considered as the “Normal” status. Otherwise it is determined as a “Breaking” status (*i.e.*, as three axial data are continuously checked in a roll and only if NONE of the three axial data indicates the “Normal” status, the current status is determined as the “Breaking” status).

In this criterion, the threshold voltages are set as user-defined experimental values which can be obtained and defined by the users in an individual separate cutter-breakage milling test with the same milling parameters/conditions (Note: breakage conditions are easy to observe in any actual milling situation and thus the threshold voltage values are easily defined by the users). Through the criterion, the accuracy of monitoring the cutter breakage is 100%. These results show that our approach of monitoring a cutter breaking during milling is successfully validated. In addition, according to the criterion established from cutter-wear experiment, both the cutter-breakage and the moment when a cutter is about to break can also be monitored. This monitoring of cutter-breakage caused by gradual wear is complementary to the above monitoring of cutter-breakage caused by unexpected conditions. With both monitoring solutions, the milling-processes and cutter-wear/breakage-condition monitoring abilities of our sensing system are more complete. Finally, we also note that at this moment, the sensing function of the system may be slightly influenced when operating at high speeds. This is because that the cutting force is decreased as the spindle speed is increased (while the rest of the milling conditions remain the same) [53]. Therefore, a lower cutting force causes a milling signal with lower magnitude. Consequently, the lower magnitude of the milling signal causes a lower signal-to-noise ratio. This may become a problem to distinguish the signals from the noises. To address this noise issues at high speed, noise filtering should be used for signal preprocessing in the future.

Finally, we also notice that more detailed investigation of variation and influence of several milling factors would enhance our system’s demonstration. However, this will also significantly diverge from the main focus of our approach, which was to demonstrate a new approach for using an attachable energy harvester to drive a wireless vibration sensing system to monitor milling-process and cutter-wear/breakage conditions, rather than a detailed analysis of milling/cutting-monitoring results. A similar claim is also reported in [21]. They reported that “*The focus of the case study was not on vibration analysis per se. Rather, experiments were carried out to show that wireless sensor networks, and their individual wireless sensor platforms, could provide new tools for research in predictive maintenance and condition-based monitoring of factory machinery in general …*”. Due to the claim and the technical contents of this representative article, we think that the initial/preliminary analysis of the results in this paper is sufficient and thus our approach offers a new tool for users to apply to most general milling-processes and cutter-wear/breakage condition monitoring.

## 5. Conclusions

An attachable electromagnetic-energy-harvester driven wireless vibration-sensing system to monitor milling-processes and cutter-wear/breakage-conditions is successfully demonstrated. The results show the system is capable of sensing vibrations occurring during milling. Furthermore, through the criteria established from analyzing the experimental vibration signals, the system can successfully simulate the operating sequences of the milling processes and monitor a cutter’s health-conditions during milling (such as cutter-wear conditions and cutter-breakage occurrence). These results show that the attachable energy-harvester-driven intelligent wireless machining-monitoring intelligent system is successfully achieved. Consequently, the corresponding milling-processes and cutter-wear/breakage-conditions monitoring approaches can be further modified to apply to general milling duties of most rotational spindle-based milling machines in factories (for example, we demonstrated the application of a similar but more rough approach to another milling machine [49]). Moreover, to fit many different milling machines, each part of the system should be as compact/miniaturized as possible. In the future, we plan to develop a non-destructive, attachable/stick-on, and miniaturized/compact system which can be easily applied onto many different milling machines.

## Figures and Tables

**Table 1:** The power-budget analysis of the system.

Energy Harvesting (Power Generation) *							
Parameter	Electromagnetic Energy Harvester						Total
V (Volt)	8.19						-
I (mA)	28.1						-
P = I*V (mW)	230.14						230.14
**Power Consumption**							
Parameter	Voltage Regulator (LM7806)	Voltage Dropdown Diodes	Differential Amplifier	Voltage Regulator (LM1086)	Accelerometers	Wireless Module	Total
V (Volt)	ΔV = 2.19	ΔV = 0.66	5.34	ΔV = 2.04	3.3 for each	3.3	-
I (mA)	27.7	27.2	0.4	26.7	0.45 for each	25.35	-
P = I*V (mV)	60.66 **	17.95 **	2.14	54.47 **	4.47 ***	83.66	223.35

* The system is operated with the spindle rotational speed of 1650 rpm, the power is measured after bridge rectifier ** Power consumptions including thermal dissipation *** Total power consumption of three accelerometers

**Table 2:** The rotational-speed-dependent milling parameters/conditions for the milling-processes monitoring.

Milling Parameter/Condition							
General Milling Condition		Cutter Specification		Work Piece Specification		Accelerometer Specification	
Parameter	Value/ Condition	Parameter	Value/ Condition	Parameter	Value/ Condition	Parameter	Value/ Condition
Spindle Speed	1650 rpm	Manufacturer	Nachi- Fujikoshi Corp.	Standard Grade	JIS-SS41	Product Model	Analog Device ADXL 103
Cutting Speed	41.46 m/min	Product Model	GSX4C- 2.5D	Material	Carbon- Steel	Measurement Range	±1.7 g
Table Feed Rate	283.55 mm/min	Cutter Type	End-Mill	Size	50*50*50 (mm^3^)	Sensitivity	1000 mV/g
Depth of Cut (mm)	2	Cutter Diameter	8 mm	Milling Type	Slot Milling	Noise Density	110 µg/√Hz
Cutting Direction	x-axis	Number of Teeth	4 teeth	Milling Length (one duty-cycle)	100 mm	Resolution	1 mg at 60 Hz
Cutting Fluid	none	-	-	-	-	Resonant Frequency	5.5 kHz
